# Assessment of Stress Distribution Around Traditional and Sleeve Fixed Partial Denture Designs: Finite Element Analysis

**DOI:** 10.1055/s-0044-1791761

**Published:** 2024-12-10

**Authors:** Mohiddin R. Dimashkieh, Salah A. Yousief, Amir M. Demachkia, Mohammad Zakaria Nassani, Abdulaziz Samran, Ali Barakat, Yash Pal Singh, Reda M. Dimashkieh, Hend Mohamed El Sayed, Rami M. Galal, Mohammed Noushad, Monika Saini

**Affiliations:** 1 Department of Restorative and Prosthetic Dental Sciences, College of Dentistry, Dar Al Uloom University, Riyadh, Saudi Arabia; 2 Department of Crown and Bridge, Faculty of Oral and Dental Medicine, Al Azhar University, Assiut Branch, Egypt; 3 Department of Dental Materials and Prosthodontics, São Paulo State University (UNESP), São José dos Campos, Brazil; 4 Almeswak Private Clinics, Riyadh, Saudi Arabia; 5 Private Clinics, Riyadh, Saudi Arabia; 6 Department of Restorative Dentistry and Conservative Dentistry, Faculty of Dentistry, Cairo University, Manial, Cairo, Egypt; 7 Department of Fixed and Removable Prosthodontics, National Research Centre, Cairo, Egypt

**Keywords:** partial denture, finite element analysis, zirconia, E-max, Celta Duo

## Abstract

**Objectives** The aim of this research is to evaluate/compare the use of traditional versus sleeve fixed partial denture (PD) designs made from different materials on supporting structures. The comparison included three- and four-unit PD cases.

**Materials and Methods** Four finite element models are used in the research. The three-unit PD consists of the mandibular second premolar, first molar (as a pontic), and second molar. The four-unit PD includes the first premolar. The PD materials assessed were zirconia, E-max, and Celtra Duo. Bone has been simplified representing it as two cuboids. Each PD has been loaded to two cases over the pontic's central fossa: 300 N compressive, 150 N obliquely applied with 45 degrees forming 24 cases.

**Results** The three-unit traditional and sleeve PDs material change showed a slight change in cortical bone stress under vertical loading. Under oblique loading, cortical bone Von Mises stresses were higher by about 12 to 15% more than vertical loading. On the other hand, the four-unit PDs showed minor effect by changing PD material, while using sleeve design PD can reduce the cortical bone stresses up to 20% in comparison to traditional PD design. The mucosa and spongy bone were negligibly affected by changing PD material, and the traditional and sleeve designs showed close values to each other. Superiority of sleeve design appeared by reducing cement layer stresses dramatically, while PD body material rigidity affects its response.

**Conclusion** Within the limitations of this study, the higher rigid PD material can dissipate loadings over it more preferably regarding its effect on the underlying structures. Sleeve PD design is equivalent to the traditional one for three-unit PDs, while it showed better performance with four-unit PDs. Zirconia three-unit PDs' bodies received the lowest stresses and redistributed and transferred the applied load to the underneath structures better than the other two tested materials. This finding was reversed with four-unit PDs.

## Introduction

Complete veneer crowns are used very often as retainers in dentistry due to high retentive features if we compare it to partially veneered crowns and inlays. 1 So, full veneer restorations have been used indiscriminately in the form of retainers even on sound abutment teeth. In spite of that, a full veneer is considered destructive to the tooth structure, especially in the case of long edentulous space or when the occlusogingival height of teeth is not sufficient.

Length of abutment is a major factor in retention. Since short abutments are considered a problematic case facing prosthodontists, several attempts are made to compensate for the absence of retentive features on such abutments, mostly retention grooves, pins, and slots are employed in addition to increasing tooth length by surgery. Normally, with young patients, there is a short tooth with a low surface contacting area with retainers; teeth therefore will have insufficient retention. Abutments with low occlusogingival height were always the main reason for failed fixed prostheses. 2

Previously, the emphasis has been on less destructive dentistry. 3 4 Removing sound abutments' occlusal surfaces when preparing them is unjustified; also restoring the shape could be difficult. The prosthodontist so must be conscious of how necessary is it to conserve occlusal morphology, as normal occlusion morphology of prepared teeth helps in establishing proper occlusion records, preserving pulp integrity and maintaining the length of the crown.

The progress with resin-bonded prostheses in 1970s 1 was an advance in prosthodontics, and the effective cementation of metallic structure with perforations to enamel after etching with acid using composite cement has been described. 2 Resin-bonded prostheses with conservatively grinded teeth have been widespread accompanied by improvement of adhesion methods. 3 Resin-bonded prostheses moreover preserve the abutment remaining structure while prosthetic management. 4 5 6 7

With traditional fixed dental prostheses work, the preparation of the occlusal table in the teeth and the re-establishment of occlusal surface shape are a difficult process. Resin-bonded prostheses show many merits over commonly reduced teeth to receive full coverage or partially covered restorations regarding posterior fixed dental prostheses; the benefits are listed in the literature. 8 The most significant merit of minimal tooth preparation was decreased pulpal insult, decreased chairside time, and laboratory expenses in comparison to traditional restorations. 9 While resin-bonded prostheses were in origin considered for replacing anterior, there were also a method to substitute posterior teeth. 8 9 10 11

Many *in vivo* researches have examined resin-bonded prostheses documenting a good success rate. 9 10 11 12 Some documented *in vivo* articles had shown rates of survival of about 70% with anterior and 40% with posterior with time from about 2 to 11 years. 13

Sleeve-design prostheses have been used for tackling the problem of resin-bonded prostheses and conventional prostheses on posterior teeth. 1 2 The ring design (RD) retainer is a combination of the conventional rules regarding how abutments are prepared accompanied by the theory caring about saving occlusal morphology. Ring design is a partial veneer restoration without preparing the occlusal table. 13 Abutment reduction consists of axial preparation of buccal, palatal, mesial, and distal surfaces excluding occlusal surface. Preparation is done with an intracervical chamfer. The occlusal finish line is a featheredge, which stops about 0.5 mm away from the contacting surface of opposing cusps. Sleeve or RD retainer incorporates the primary principles of retention (two opposing axial walls), 13 resistance, structural durability, and accurate margins in addition to the principle of conservative abutment preparation.

Our research shows the comparison of missing posterior teeth restored with noninvasive fixed partial denture (FPD) and conventional FPD.

Traditional retainers with teeth having small occlusogingival height can fall off easily due to low retentive features after preparing the occlusal surface.

Recent RD of retainers with posterior replacement improving retentive features accompanied with less tooth preparation and keeping the proper occlusion is presented.

Our research aimed to assess the performance of traditional compared with sleeve partial denture (PD) design and to evaluate these PD materials in cases of three and four units.

## Materials and Methods

**Fig. 1:**
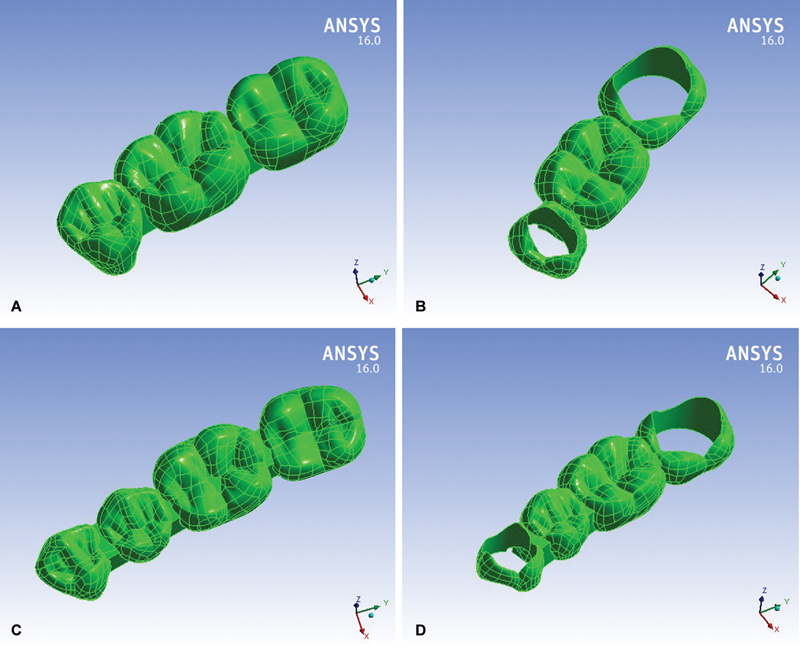
( **A–D** ) Partial denture on the four studied models.

**Fig. 2:**
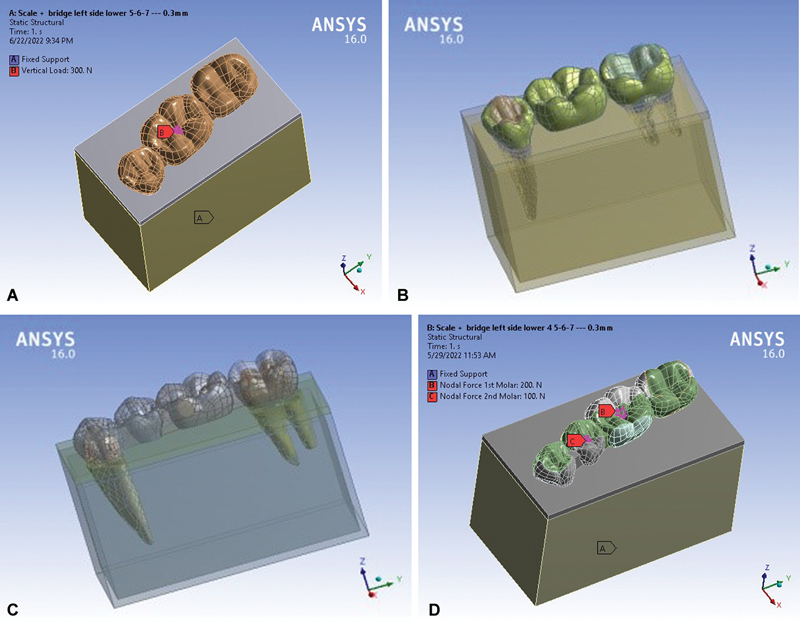
Complete models: ( **A** ) three-unit traditional, ( **B** ) three-unit sleeve, ( **C** ) four-unit traditional, and ( **D** ) four-unit sleeve.

Starting from computed tomography scan views for the complete jaw, a geometric model for the chosen four teeth was prepared. First and second premolars, and first and second molars were separated to construct the bridge. Intermediate software (3-Matic versions 15.01, Materialize, Nevada, United States) was used to cut the needed points to edit STL file errors and produce solid teeth models. The two outer abutments were done to support conventional and sleeve PDs. A connector of 3 × 3 mm was placed between the three and four teeth crowns/pontics. Bone and mucosa geometries had been made more simple and developed in the form of three cuboids, while a sponge bone cavity within a cortical bone, root, periodontal ligament (PDL), and cement (Rely X Cement) 40 μm thickness had been designed using a set of Boolean operations. Fig. 1 compares the last PDs geometry, while Fig. 2 demonstrates the final four models' geometry, (1) Model #1: three-unit traditional PD, (2) Model #2: three-unit sleeve PD, (3) Model #3: four-unit traditional PD, and (4) Model #4: four-unit sleeve PD.

All materials used within the research were dealt with as if they were isotropic, homogenous, and linearly elastic. The properties list is shown in Table 1 .

**Table 1:** Materials properties

Materials	Young's modulus (MPa)	Poisson's ratio
Cortical bone	13,700	0.30
Spongy bone	1,370	0.30
Mucosa	10	0.40
Periodontal ligament	68	0.45
Root	18,600	0.31
Cement (Rely X) (40 µm thickness)	8,000	0.30
Fixed prosthesis materials		
Zirconia	210,000	0.35
E-max	91,000	0.23
Celtra Duo	107,900	0.222

**Fig. 3:**
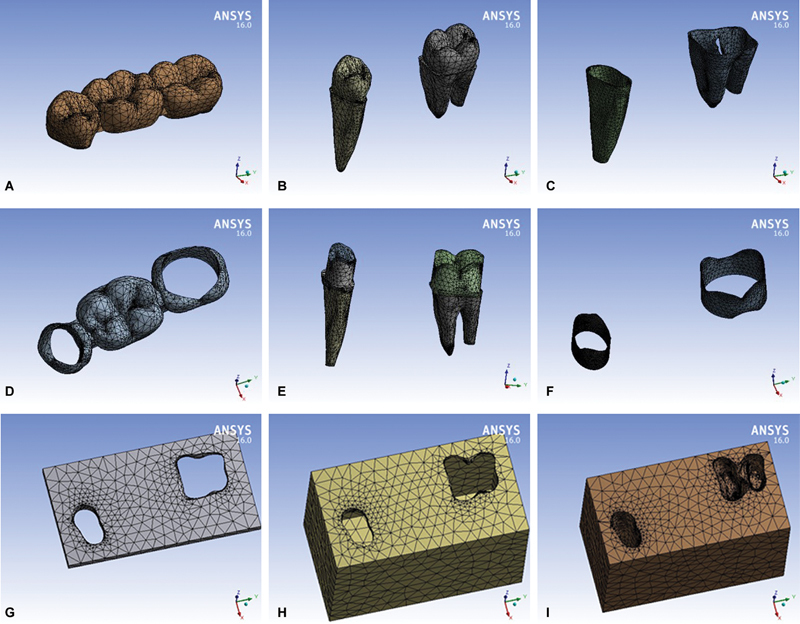
( **A–I** ) Sample of the meshed three-unit model components.

**Fig. 4:**
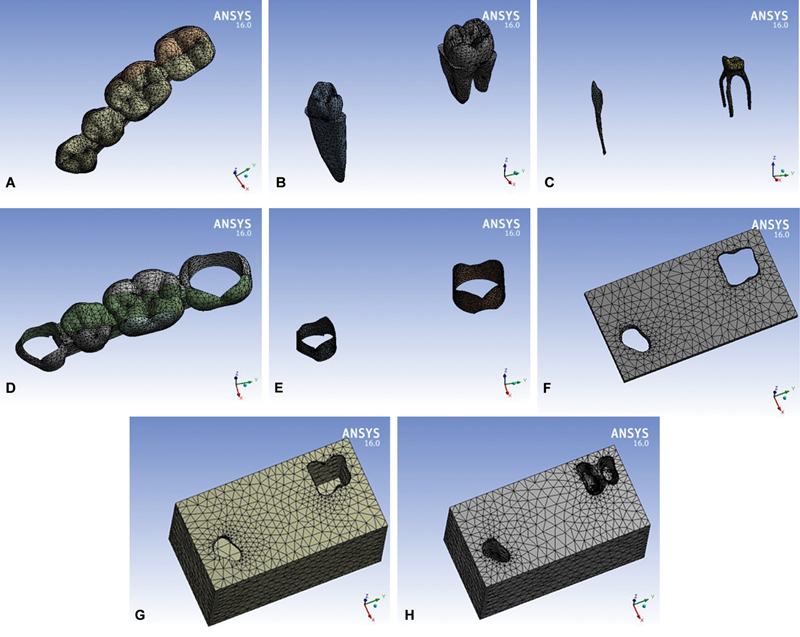
( **A–H** ) Sample of the meshed four-unit model components.

Model mesh components were created on the ANSYS Workbench environment (ANSYS Inc., Canonsburg, Pennsylvania, United States). Meshing was done using three-dimensional solid element having three degrees of freedom (translation in main axes directions). 12 Mesh density had been examined and optimum accuracy with reasonable calculation time was obtained. Resulted numbers of nodes and elements are shown in Table 2 with samples for the meshed components of the three-unit PDs shown in the form of screenshots from the ANSYS screen in Fig. 3 . Additionally, Fig. 4 demonstrates samples of the meshed components of the four-unit PDs.

**Table 2:** The used mesh density

	Model #1—three-unit traditional		Model #2—three-unit sleeve		Model #3—four-unit traditional		Model #4—four-unit sleeve	
	Nodes	Elements	Nodes	Elements	Nodes	Elements	Nodes	Elements
Cortical bone	20,943	13,011	20,943	13,011	14,300	7,125	14,280	7,118
Spongy bone	202,426	146,836	202,844	147,189	30,758	18,492	30,704	18,462
Mucosa	20,689	13,751	20,689	13,751	6,175	2,974	6,167	2,969
First premolar root	–	–	–	–	20,963	12,633	38,785	22,579
First premolar PDL	–	–	–	–	12,708	6,451	14,081	7,058
Cement layer (first premolar)	–	–	–	–	14,528	7,135	7,488	3,383
Second premolar root	200,369	145,067	63,485	44,984	–	–	–	–
Second premolar PDL	13,981	7,318	161,399	115,351	–	–	–	–
Cement layer (Second premolar)	97,650	65,534	32,486	15,971	–	–	–	–
Second molar root	203,260	147,113	160,953	116,293	35,045	21,238	28,071	16,021
Second molar PDL	39,757	24,543	39,757	24,543	21,647	11,020	21,974	11,186
Cement layer (second molar)	13,216	6,544	7,940	3,667	29,896	14,064	9,966	4,602
Partial denture	150,966	107,119	105,909	75,576	33,149	18,004	29,080	15,627

Abbreviation: PDL, periodontal ligament.

In each model, the PD was subjected to two loading cases, where the load was placed over the central fossa of pontic(s) with parameters, 300 N compression and 150 N obliquely applied load with 45 degrees forming 24 cases. The four-unit models divided the vertical (compressive) load as 200 N on the first molar and 100 N on the second premolar, while half of these values were applied in oblique cases (see loading sites in Fig. 2A–D ).

The lowest area of cortical bone cuboids has been set as fixed within the place as a boundary condition. Finite element analyses (linear static) were done on a Workstation HP Z820 (Dual Intel Xeon E5-2670 v2 processors, 2.5 GHz, 64.0 GB RAM). The resulting data of the four models were checked with other studies 13 showing an agreement.

## Results

**Fig. 5:**
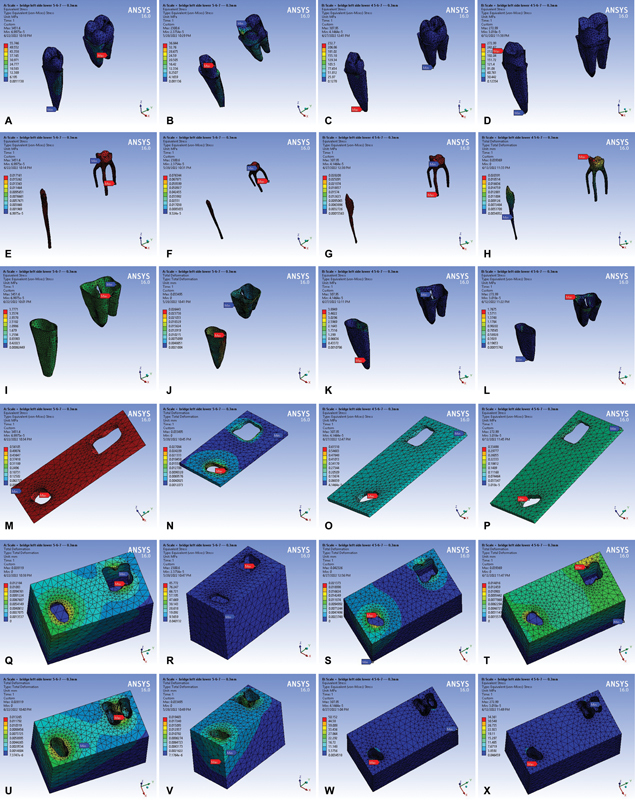
( **A–X** ) Sample of the results as screenshots from ANSYS screen.

**Fig. 6:**
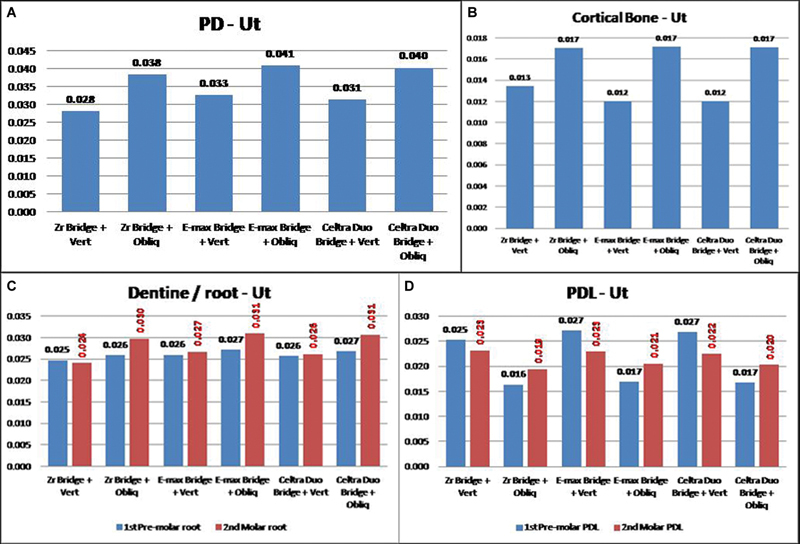
Sample of the comparison results of total deformations: ( **A** ) traditional three-unit partial denture (PD), ( **B** ) cortical bone under three-unit sleeve PD, ( **C** ) roots under four-unit traditional PD, and ( **D** ) periodontal ligament (PDL) under four-unit sleeve PD.

A huge number of graphical representations were obtained from the 24 case studies, which need large space to present. Fig. 5 gives a sample of the obtained results from the ANSYS screen. For the three-unit PD, only significant differences in comparison of extreme deformations are presented in Fig. 6 . PD body deformation ( Fig. 6A ) was slightly affected by PD (its) material. According to PD material rigidity, it showed less deformation thus deformation increased from zirconia to Celtra Duo to E-max.

On the other hand, cortical bone deformations ( Fig. 6B ) were negligibly affected by changing PD material, while its values altered by about 30% under oblique load in comparison to vertical load. Dentine (root) and PDL deformations ( Fig. 6C, D ) showed insensitive behavior to changing PD material and small changes with changing load direction (from vertical to oblique). Contrarily, the four-unit PD did not show significant differences in deformations, where minor or negligible effects can be noticed related to PD material rigidity or PD design.

**Fig. 7:**
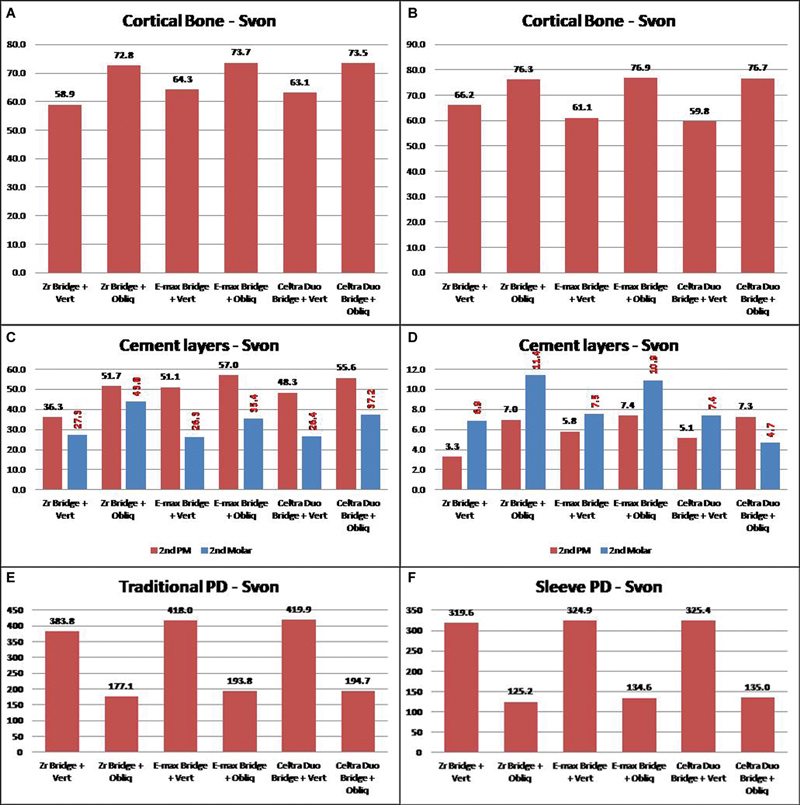
( **A–F** ) Sample of the results comparison of Von Mises stress for the three-unit partial denture (PD).

**Fig. 8:**
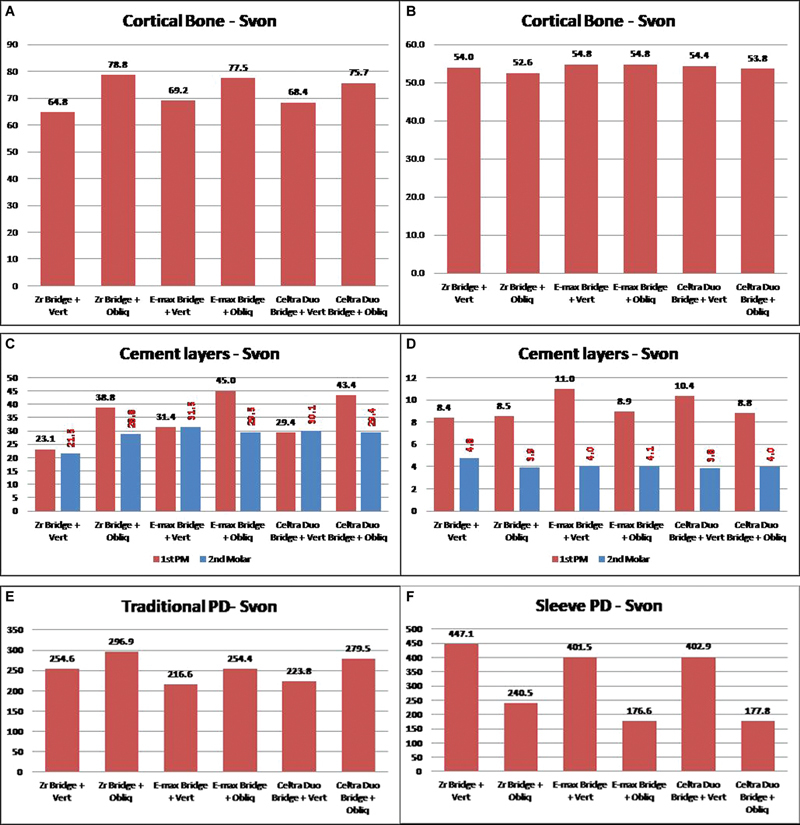
( **A–F** ) Sample of the results comparison of Von Mises stress for the four-unit partial denture (PD).

- Von Mises stresses showed more variations with PD design or material than deformation. Fig. 7A, B shows a comparison of cortical bone extreme Von Mises stresses that appeared on three-unit traditional and sleeve PDs, respectively. Changing PD material showed a slight change in cortical bone stress under vertical loading, and the PD material rigidity trend was reflected in the Von Mises stress. While the obliquely applied load value was half of the vertically applied one, Von Mises stress was higher by about 12 to 15% more than those obtained by vertical loading. The two PD designs showed nearly equivalent values; thus, the PD design can be exchanged according to the patient's case.
- The mucosa and sponge bone were negligibly affected by changing PD material, and the traditional and sleeve designs showed close values to each other.
- PDL, pulp, and remaining tooth (dentine + enamel) showed insensitive behavior to PD material, while the remaining tooth was receiving more stresses under sleeve design by about 15%.
- Cement layers comparison in Fig. 7C, D showed considerable changes in stress values under different PD materials, which showed the superiority of using zirconia as a PD material, then Celtra Duo, and finally, E-max. The sleeve PD design generated very small values of stresses on the cement layer and the difference may reach 75% less than the traditional PD design.
- PD body stresses comparison ( Fig. 7E, F ) showed that zirconia as PD material received the lowest stresses, while E-max and Celtra Duo showed similar behavior with very close values of deformations and stresses, where sleeve design received less stress than traditional PD design. Four-unit PDs also showed considerable variation in extreme Von Mises stress values as presented in Fig. 8 . Cortical bone in Fig. 8A, B is slightly affected by changing PD material, while using sleeve design PD can reduce the stresses up to 20% in comparison to traditional PD design.
- Again, the mucosa and spongy bone were negligibly affected by changing PD material, and the traditional and sleeve designs showed close values to each other. PDL, pulp, and remaining tooth (dentine + enamel) showed insensitive behavior to PD material, while all of them were receiving more stresses under sleeve design by about 15% under vertical load. Contrarily, all of them were receiving less stress under sleeve design by about 15% under oblique load.
- Cement layers ( Fig. 8C, D ) recorded small changes in stress values under different PD materials, which showed the superiority of using zirconia as a PD, then Celtra Duo, and finally, E-max. The sleeve PD design generated very small values of stresses on the cement layer and the difference may reach 60% less than the traditional PD.
- The long (four units) PD body ( Fig. 8E, F ) showed a gradual decrease in its stresses using less rigid material.

## Discussion

### Three-Unit FPDs

Cortical bone deformations were negligibly affected by PD material, while its values altered by about 30% under oblique load in comparison to vertical load. This finding may refer to load energy dissipation inside model components, thus minor energy (effect) reached the cortical bone. In a study by Mohamed et al in 2022, it was stated that different materials used for prosthetic replacements showed comparable results regarding stresses induced by them. 1

Although the changes in cortical bone stresses were relatively small, it kept one trend: its stresses decreased with increasing PD material rigidity. PD design change did not show a significant effect, that is, both designs can replace each other. Also, Ishak et al in 2022 found that with decreasing the implant stiffness, there is an increase in the transferred stresses to the surrounding structures. 2

Sleeve PD design reduces cement stresses in comparison to traditional one, and typically PD rigidity increase resulted in reducing the cement layer stresses. This was approved with Yousief et al in 2022 in a finite element study showing also that using more rigid prosthetic material led to decreasing the stresses falling over the cement layer.

PD body deformation is slightly affected by its material. That PD material rigidity increase will reduce deformation thus deformation increased from zirconia to Celtra Duo to E-max. This was augmented by Kasem et al in 2023 who found that the more the rigidity of the material used in fixed prostheses, the less the deformations and catastrophic failures of this material with stresses, and the more stresses are transferred to the underlying and supporting structures. 4

Zirconia PD in both designs showed less deformation and stresses, while Celtra and E-max were nearly equivalent. This behavior was expected due to having very close properties. Mohamed et al also proved this in 2022 showing that laminate veneers fabricated from E-max and Celtra duo showed nonsignificant difference in their reaction to stresses regarding fracture resistance. 5

### Four-Unit FPDs

Dissipation of applied load energy in the large PD was recognized as total deformation and showed minor or negligible effect with different PD material or design in all four-unit model parts except the PD body. PD body deformed less with increasing PD material rigidity. Schwindling et al augmented this in 2022 and found that with the use of long-span bridges especially with rigid materials such as zirconia, the deformation of the material and the stresses transfer to the underlying structures are reduced. 6

From a cortical bone point of view, PD material did not have a significant effect, while sleeve PD design can be used to reduce cortical bone stresses. On the contrary, Yu et al in 2023 found that zirconia material reduced the stresses on a cortical bone, especially with full arch frameworks. 7

Cement lifetime will increase under the higher rigid material; similarly, using sleeve design will reduce the cement layers stresses. Thus, for best practice, it is recommended to use sleeve zirconia PD. On the contrary, Sukumoda et al in 2021 stated that stresses on the cement line and its lifetime are reduced with increasing the surface area of the prosthetic design of the bonding surface area. 8

Four-unit PD body showed a gradual decrease in its stresses using less rigid material. That matches many literature-correlated PD behaviors and their material rigidity. Tribst et al in 2019 stated that using resin composite material as a prosthetic material for a fixed dental prosthesis with low rigidity decreased the stresses on the cement and the prosthetic material itself. 9

The mucosa and spongy bone were negligibly affected by changing PD material, and the traditional and sleeve designs showed close values to each other. All PDs showed dentine (remaining tooth: dentine + enamel), pulp, and PDL deformations were insensitive to changing PD material. Small changes in stress values might appear with changing load direction (vertical/oblique). In addition, the remaining tooth received more stresses under the sleeve design by about 15%, and this may be explained due to less prosthetic surfaces receiving stresses. Contrarily, the four-unit PD received less stresses under sleeve design by about 15% under oblique load. In a study by Shash et al in 2023, it was found that some prosthetic materials such as the polyetheretherketone affect the spongy bone showing low stresses, while it showed high stresses on the mucosa. 10

## Conclusion

- Cortical bone showed minor differences in deformation and stresses with smallest value with the higher rigidity PD material and sleeve design.
- Mucosa and spongy bone are not sensitive to changing PD materials, while using sleeve design slightly reduces their stresses in case of three-unit PD and increases it in case of four-unit PD.
- Within the limitations of this study, the higher rigid PD material can help in distributing loads better to the underlying structures. Sleeve PD design is equivalent to the traditional one for three-unit PDs, while it showed better performance with four-unit PDs.
- Zirconia PDs' bodies received the lowest stresses, redistributed, and transferred the applied load to the underneath structures better than the other two tested materials.

## References

[1] Mohamed A MA Askar M G El Homossany M EB Stresses induced by one piece and two piece dental implants in All-on-4® implant supported prosthesis under simulated lateral occlusal loading: non linear finite element analysis study BMC Oral Health 2022 22 01 196 35599323 10.1186/s12903-022-02228-9 PMC9125928

[2] Ishak M I Daud R Noor S NFM Khor C Y Roslan H Assessment of stress shielding around a dental implant for variation of implant stiffness and parafunctional loading using finite element analysis Acta Bioeng Biomech 2022 24 03 147 159 38314490

[3] Yousief S A Mohammed R Almadani Y Assessment of stress distribution around bridge abutments (implant and natural tooth): FEA EC Dental Science 2022 21 12 40 49

[4] Kasem A T Elsherbiny A A Abo-Madina M Tribst J PM Al-Zordk W Biomechanical behavior of posterior metal-free cantilever fixed dental prostheses: effect of material and retainer design Clin Oral Investig 2023 27 05 2109 2123 10.1007/s00784-022-04813-2 PMC10160170 36456895

[5] Mohamed E Elbasty R Elshehawi D Evaluation of fracture resistance of two laminate veneers ceramic materials at two loading angulations (in-vitro study) Egypt Dent J 2022 68 2755 2764

[6] Schwindling F S Bechtel K N Zenthöfer A Handermann R Rammelsberg P Rues S In-vitro fit of experimental full-arch restorations made from monolithic zirconia J Prosthodont Res 2022 66 02 258 264 34305088 10.2186/jpr.JPR_D_20_00321

[7] Yu W Chen S Ma L Ma X Xu X Biomechanical analysis of different framework design, framework material and bone density in the edentulous mandible with fixed implant-supported prostheses: a three-dimensional finite element study J Prosthodont 2023 32 04 309 317 35546271 10.1111/jopr.13532

[8] Sukumoda E Nemoto R Nozaki K Increased stress concentration in prosthesis, adhesive cement, and periodontal tissue with zirconia RBFDPs by the reduced alveolar bone height J Prosthodont 2021 30 07 617 624 33219705 10.1111/jopr.13293

[9] Tribst J PM Dal Piva A MO de Melo R M Borges A LS Bottino M A Özcan M Short communication: Influence of restorative material and cement on the stress distribution of posterior resin-bonded fixed dental prostheses: 3D finite element analysis J Mech Behav Biomed Mater 2019 96 279 284 31077955 10.1016/j.jmbbm.2019.05.004

[10] Shash Y H El-Wakad M T El-Dosoky M AA Dohiem M M Evaluation of stresses on mandible bone and prosthetic parts in fixed prosthesis by utilizing CFR-PEEK, PEKK and PEEK frameworks Sci Rep 2023 13 01 11542 37460592 10.1038/s41598-023-38288-2 PMC10352298

[11] Kohnke P ANSYS Mechanical APDL Theory Reference Canonsburg, PA ANSYS Inc. 2013

[12] Attia M A Effect of material type on the stress distribution in posterior three-unit fixed dental prosthesis: a three-dimensional finite element analysis Egypt Dent J 2018 64 04 3907 3918

[13] Yousief S A Ateeq J Alsubhi A Finite element study on posterior three-unit fixed dental prosthesis made from different materials EC Dental Science 2020 19 37 43

